# Propolis Modulates the Gut Microbiota–Gut Hormone–Liver AMPK Axis to Ameliorate High-Fat Diet-Induced Metabolic Disorders in Rats

**DOI:** 10.3390/nu17193114

**Published:** 2025-09-30

**Authors:** Yanru Sun, Wanwan Huang, Yingying Shang, Mohamed G. Sharaf El-Din, Hua Hang, Peng Wang, Cuiping Zhang, Yuan Huang, Kai Wang

**Affiliations:** 1 College of Life Sciences, Anhui Normal University, Wuhu 241000, China; sunyanru@ahnu.edu.cn (Y.S.); huangwanwan1111@163.com (W.H.); syy200017@163.com (Y.S.); 2006hanghua@163.com (H.H.); wangpeng@ahnu.edu.cn (P.W.); 2 State Key Laboratory of Resource Insects, Institute of Apicultural Research, Chinese Academy of Agricultural Sciences, Beijing 100093, China; mohamed.sharaf@pharm.psu.edu.eg (M.G.S.E.-D.); wangkai@caas.cn (K.W.); 3 Pharmacognosy Department, Faculty of Pharmacy, Port Said University, Port Said 42515, Egypt; 4 College of Animal Sciences, Zhejiang University, Hangzhou 310058, China

**Keywords:** propolis, gut hormones, gut microbiota, AMPK signaling, lipid metabolism

## Abstract

**Objectives**: Emerging evidence suggests that propolis possesses significant anti-obesity properties. While gut hormones and microbiota are known to play crucial roles in obesity development, the specific mechanisms through which propolis exerts its effects via the gut hormone axis remain poorly characterized. **Methods**: A high-fat diet (HFD) rat model was established to investigate the regulatory effects of propolis. After 10 weeks of intervention, blood serum, liver, colon tissues, and luminal contents were analyzed for metabolic parameters, gene expression of gut hormones and AMPK pathway markers, microbial community structure, and short-chain fatty acid production. **Results**: Propolis effectively mitigated HFD-induced metabolic disturbances, including excessive weight gain, adipose tissue accumulation, hyperlipidemia, and hepatic dysfunction. These improvements were associated with significant upregulation of the AMPK pathway. Importantly, propolis enhanced intestinal barrier integrity and differentially modulated gut hormone expression by increasing the mRNA levels of *Cck*, *Gip*, and *Ghrl*, and decreasing *Lep* and *Gcg* levels. 16S rRNA sequencing analysis revealed that propolis administration selectively enriched butyrate- and propionate-producing bacterial species. Correlation analysis further identified the *Eubacterium brachy* group as a pivotal microbial mediator in the propolis-modulated gut microbiota–gut hormone–liver AMPK axis. **Conclusions**: Our findings establish that propolis ameliorates obesity-related metabolic disorders by orchestrating crosstalk among gut microbiota, enteroendocrine hormones, and hepatic AMPK signaling. These results elucidate a novel mechanistic pathway in rodents; however, their direct translatability to humans requires further clinical investigation. This tripartite axis offers a mechanistic foundation for developing microbiota-targeted anti-obesity therapies.

## 1. Introduction

Chronic high-fat diet (HFD) induces excessive fat accumulation, which ultimately leading to dyslipidemia and subsequent metabolic dysfunction [1]. These metabolic disorders, including obesity, insulin resistance, and elevated triglycerides, increase the risk of severe health complications such as type 2 diabetes and cardiovascular diseases. While Glucagon-like peptide-1 (GLP-1) receptor agonists (e.g., *Semaglutide*) show promise in weight loss and lipid reduction by mimicking gut hormones [2], their high cost and side effects limit accessibility. This limitation has spurred increasing interest in natural alternatives that can similarly target gut hormone-mediated metabolic regulation, offering a potentially safer and more affordable approach to managing high-fat diet-induced metabolic disorders. Propolis, a natural product with documented metabolic benefits, emerges as a promising candidate. However, while its effects on gut microbiota and AMPK signaling have been explored, its specific role in modulating gut hormones—a key nexus in metabolic regulation—remains poorly characterized, representing a critical knowledge gap and the primary novelty of this investigation.

Gut hormones, including GLP-1, GIP, glucagon (GCG), and ghrelin (encoded by the gene *Ghrl*), are secreted by enteroendocrine cells (EECs) and play critical role in regulating energy homeostasis, food intake, and lipid metabolism [3]. Their effects are partly mediated by AMP-activated protein kinase (AMPK), a central metabolic sensor that modulates lipid synthesis (via sterol regulatory element-binding factor (SREBF)-1/2, fatty acid synthase (FASN)), and oxidation via peroxisome proliferator-activated receptors (PPARs)/acetyl-CoA carboxylase alpha (ACC1, encoded by the gene *Acaca*)) [4,5,6]. Intriguingly, gut hormones exhibit bidirectional crosstalk with gut microbiota: microbial metabolites, such as short chain fatty acids (SCFAs) and bile acids, stimulate EECs to secrete hormones, which in turn influence hepatic AMPK activity and lipid metabolism [7,8,9]. Furthermore, accumulating evidence highlights a bidirectional complex interaction between gut microbiota and gut hormones [10,11]. For instance, microbial metabolites (e.g., SCFAs) stimulate EECs to secrete hormones to inhibit hepatic FASN expression and PPAR*α* and PPARγ [12,13]. This intricate gut microbiota–gut hormone–liver AMPK axis represents a promising therapeutic target. Given propolis’s known interactions with both gut microbiota and AMPK signaling, we hypothesized that it may exert its lipid-lowering effects by orchestrating crosstalk within this axis, with a particular focus on its unexplored capacity to modulate gut hormone expression.

Propolis is a resinous substance collected by honeybee (*Apis mellifera*) from various plant sources, mixed with wax and bee secretion, and used in hive construction, rich in polyphenols and other bioactive compounds, which vary significantly depending on the botanical and geographical origin. This complex chemical composition has garnered attention for its diverse biological activities, including anti-inflammatory, antioxidant, antibacterial, and antiviral properties making propolis a promising natural therapeutic agent [14,15]. Recent studies have highlighted its potential in modulating lipid metabolism and intestinal health, further positioning it as an attractive option for improving metabolic health [16]. For instance, propolis downregulates hepatic lipogenic genes in AMPK signal pathway including *Srebf-1/2*, *Acaca*, and *Fasn* in obese mice [17], while upregulating *Pparγ* to ameliorate adiposity [18]. Additionally, propolis influences gut microbiota composition, enhancing microbial diversity and restoring SCFAs production in HFD-fed mice [19,20]. It also strengthens intestinal tight junctions via the AMPK/ERK pathways [21]. Notably, bioactive components of propolis, such as ferulic acid, stimulate the secretion of intestinal hormones such as cholecystokinin (CCK) and GLP-1 in Caco-2 cells [22] and correct the leptin/ghrelin imbalance in obesity [23]. However, these valuable insights are primarily derived from component-level or in vitro studies. A systematic, in vivo investigation into whether whole-propolis supplementation modulates lipid metabolism by directly regulating the gut hormone axis is lacking.

Accordingly, it was hypothesized that propolis ameliorates HFD-induced dyslipidemia by modulating the gut microbiota–gut hormone axis to activate hepatic AMPK signaling. To test this hypothesis, a rat model of metabolic disorder was established through a high-fat diet. The regulatory effects of propolis on lipid metabolism disorders were assessed through daily observations and physical indicators. Based on the correlation analysis between gut microbiota and gut hormones, the study identified gut microbes susceptible to gut hormones. The aim of this study is to explore the synergistic effects and potential mechanisms of propolis on gut microbiota–gut hormone–hepatic AMPK pathway–liver lipid metabolism remodeling.

## 2. Materials and Methods

### 2.1. Preparation of Propolis

Propolis used in this study was poplar type propolis, which was produced by the *Apis mellifera* honeybees. The collection and ethanol extraction were kindly provided by Fujian Shenfeng Technology Development Co., Ltd. (Fuzhou, China). The total phenolic acid content of the propolis ethanol extract was 120.3 ± 0.8 (GAE mg/g), and the total flavonoid content was 65.4 ± 1.7 (mg/g). The freeze-dried propolis ethanol extract was rapidly ground using a mortar and pestle until a fine powdered form was obtained and stored at −20 °C for subsequent use.

### 2.2. Animal Experimental Design and Procedures

A total of 24 male Sprague-Dawley rats (200 ± 10 g, 4–5 weeks old, specific pathogen free grade) were purchased from Changzhou Cavens Experimental Animal Co., Ltd. (Changzhou, China). After one week of acclimatization, the rats were randomly divided into four groups (*n* = 6 per group) using a computer-generated random number sequence: the Normal Control (NC) group; the HFD group; the low dose of propolis group, i.e., 150 mg/kg Propolis (P150) group; the high dose of propolis group, i.e., 300 mg/kg Propolis (P300) group. We use these doses with reference to the previous reports [20,24,25] and combined with the results of the preliminary experiment. Propolis was suspended in a 0.5% sodium carboxymethyl cellulose (CMC-Na) solution to achieve 25 mg/mL (for P150 group) and 50 mg/mL (for P300 group) following our established method [26]. The NC group and HFD group received 0.5% CMC-Na solution with a volume of 10 µL/g of body weight daily via gavage, while the P150 group and P300 group received 150 mg/kg and 300 mg/kg propolis via the same volume and method, respectively. The HFD group, P150 group, and P300 group were fed with a 60% high- fat diet (XTHF60, Jiangsu Xietong Pharmaceutical Bio-Engineering Co., Ltd., Nanjing, China) [27], while the NC group was fed a standard maintenance diet (XTCO1GY-001, Jiangsu Xietong Pharmaceutical Bio-Engineering Co., Ltd.). The animals were housed two per cage under controlled environmental conditions, including a room temperature of 24 ± 2 °C, relative humidity of 40–60%, and a 12 h light/dark cycle. All animals were allowed free access to food and water throughout the experiment. Food intake and water consumption were measured every two days. Specifically, a pre-weighed amount of diet was provided to each cage, and the remaining food (including any spillage collected from the cage bottom) was weighed after 48 h. The average daily food intake per rat was then calculated. Similarly, water consumption was monitored by weighing the water bottles at the beginning and end of each 48 h period. Body weight was also recorded every two days for 10 consecutive weeks.

At the end of the experimental period, the rats were humanely euthanized using CO_2_ asphyxiation. Blood samples were collected from the abdominal aorta and centrifuged at 4000 rpm for 15 min using an LC-8S centrifuge (JOANLAB Equipment Co., Ltd., Huzhou, China) to obtain serum. Subsequently, biological samples, including liver tissue, colon tissue, and colonic contents, were carefully dissected and stored at −80 °C for further analysis.

To ensure methodological rigor and minimize bias, a blinding procedure was implemented during the outcome assessment phase. The investigators responsible for the histological evaluations and molecular analyses were blinded to the group allocation of the samples throughout the data collection and analysis process. The random allocation sequence was generated by an independent researcher not involved in the endpoint measurements.

All experimental procedures were approved by the Academic Ethics Committee of Anhui Normal University (Approval No. AHNU-ET2022013).

### 2.3. Inclusion and Exclusion Criteria

All criteria for the inclusion of animals and experimental units, as well as for the exclusion of data points, were established a priori prior to the initiation of data collection and analysis. No animals or data points were excluded post hoc based on experimental outcomes. All animals that successfully completed the experimental protocol without any unforeseen complications, such as mortality from non-experimental causes or severe illness, were included in the study, yielding a final cohort of six biological replicates per group for the assessment of fundamental physiological endpoints.

Consequently, serum biochemical parameters and histopathological examinations of liver and colon tissues were performed on all six replicates per group to ensure a comprehensive evaluation of individual physiological and morphological states. For molecular analyses, a stratified sampling approach was employed to rationally allocate resources while maintaining statistical power. A random subset of four animals per group was selected for RNA extraction and subsequent qPCR analysis using a computer-based random number generator. Only RNA samples meeting pre-defined quality control standards (OD260/280 ratios between 1.8 and 2.0) were processed further.

Furthermore, for the high-throughput, cost-intensive 16S rRNA sequencing and targeted metabolomics analyses, a second random subset of three biological replicates per group was selected. This sample size was determined a priori and is consistent with established practices in microbiome and metabolomics research, where the depth of data generated per sample compensates for a lower number of biological replicates [28,29]. All samples processed for sequencing and metabolomics were subject to and passed platform-specific quality control measures prior to inclusion in the final dataset.

### 2.4. Serum Biochemical Analysis

Serum parameters including total cholesterol (TC), triglycerides (TG), and low-density lipoprotein cholesterol (LDL-C) were evaluated by total cholesterol assay kit (cat. no. A111-1-1), triglycerides assay kit (cat. no. A110-1-1) and low-density lipoprotein cholesterol assay kit (cat. no. A113-1-1) following the manufacturer’s instructions (Nanjing Jiancheng Bioengineering Institute, Nanjing, China). Additionally, serum aspartate aminotransferase (AST), alanine aminotransferase (ALT), and alkaline phosphatase (AKP) activities were assessed by aspartate aminotransferase assay kit (cat. no. C010-2-1), alanine aminotransferase assay kit (cat. no. C009-1-1), and alkaline phosphatase assay kit (cat. no. A059-1-1) according to the manufacturer’s instructions (Nanjing Jiancheng Bioengineering Institute, China). All samples from each group (*n* = 6) were analyzed.

### 2.5. Histopathological Examination of Liver and Colon Tissues

Liver and colon tissues from all animals that completed the experimental protocol (*n* = 6 per group) were collected for histopathological assessment. Liver and colon histopathology was assessed via hematoxylin and eosin (H&E) staining following a previous reported procedure [30] with several modifications. Briefly, fresh liver and colon tissues were immediately fixed in 4% paraformaldehyde solution (Biosharp Life Sciences, Hefei, China) for 48 h at 4 °C, followed by 12 h of rinsing running with water to remove residual fixative. The tissues were then sequentially dehydrated through a graded ethanol series (50%, 70%, 80%, 95%, and absolute ethanol; 1 h per concentration). Subsequently, the tissues were cleared in xylene and infiltrated with paraffin through three changes (Phygene, Fuzhou, China): first in a 1:1 paraffin/ethanol mixture (30 min), then in pure paraffin (15 min), followed by two additional paraffin baths (30 min and 20 min, respectively). The processed tissues were embedded in paraffin blocks and sectioned at a 5 μm thickness using a rotary microtome (Leica Microsystems GmbH, Wetzlar, Germany). After standard deparaffinization and rehydration, sections were stained with hematoxylin and eosin, dehydrated through an ethanol series (70%, 80%, 95%, and 100%), cleared in xylene, and mounted with neutral balsam. Histological evaluation was performed under a light microscope (Nikon Eclipse E100, Tokyo, Japan), and representative images from each group were captured for presentation. Hepatic steatosis and inflammation were assessed on H&E-stained sections by a pathologist blinded to the groups according to the nonalcoholic fatty liver disease activity score (NAS) as previously described. The total score for each liver sample ranged from 0 to 8 [31]. We focus on the condition of edema in intestinal structures, describing it based on the degree of edema.

### 2.6. Quantification of Hepatic and Colonic Gene Expression

Total RNA was extracted from liver tissues and colon tissues of a randomly selected subset of animals (*n* = 4 per group) using TRIzol reagent (cat. no. HRN0144, Herui Biotechnology, Fuzhou, China). RNA quantification was performed on a Spark multimode microplate reader (Tecan Group Co., Ltd., Männedorf, Switzerland). Only RNA samples exhibiting OD260/280 ratios between 1.8 and 2.0 were used for subsequent experiments, with a standardized input of 2 μg per reaction as described before [32]. Thereafter, the cDNA synthesis and quantitative real-time PCR analysis were performed following the instructions [33]. The cDNA synthesis completed by a reverse transcription kit (cat. no. HRF0182, Herui Biotechnology, Fuzhou, China). Quantitative real-time PCR (qPCR) was performed using the HRbio™ SYBR Green Master Mix (No Rox) (cat. no. HRF0032, Herui Biotechnology, Fuzhou, China), and the CFX96 real-time PCR system (Bio-Rad Laboratories, Hercules, CA, USA) was used to determine the relative expression levels of the genes, with three independent replicates for each sample. The relative expression levels of the target genes were calculated using the 2^−ΔΔCt^ method. Gene-specific primers were designed using NCBI Primer-BLAST and existing literature, with sequences provided in Table S1.

### 2.7. 16S rRNA High-Throughput Sequencing

Colonic content microbial genomic DNA was extracted from three randomly selected biological replicates per group (*n* = 3), processed, and sequenced by Bio-Tree Biomedical Technology Co., Ltd. (Shanghai, China). Briefly, the genomic DNA of the colonic content was extracted using TGuide S96 Magnetic Soil/Stool DNA Kit (Tiangen Biotech (Beijing, China) Co., Ltd.) according to manufacturer’s instructions. The V1-V9 hypervariable regions of the 16S rRNA gene were amplified using primers 27F (AGRGTTTGATYNTGGCTCAG) and 1492R (TASGGHTACCTTGTTASGACTT). The amplicons were quantified, after which the normalized equimolar concentrations of amplicons were pooled and sequenced on the PacBio Sequel II platform (Shanghai Biotree Biomedical Technology Co., Ltd., Shanghai, China).

Bioinformatic analysis was performed as follows. Raw reads were processed using SMRT Link software (version 8.0) to obtain circular consensus sequencing (CCS) reads, which were then demultiplexed and subjected to quality control (including primer removal and length filtering) using Cutadapt (version 2.7). Chimeric sequences were removed with the UCHIME algorithm. The resulting high-quality sequences were clustered into operational taxonomic units (OTUs) at a 97% similarity threshold using USEARCH (version 10.0), and low-abundance OTUs (counts < 2) were filtered out.

### 2.8. Targeted Metabolomics of SCFAs

SCFAs in colonic content from three randomly selected biological replicates per group (*n* = 3) were quantified. The colon contents were homogenized with 1 mL of ultrapure water by vortexing for 10 s. Subsequently, steel beads were added, and the mixture was ground at 40 Hz for 4 min, followed by three cycles of ultrasonic crushing (5 min each) in an ice-water bath. After centrifugation (4 °C, 5000 rpm, 20 min), 0.8 mL of supernatant was collected and acidified with 0.1 mL of 50% H_2_SO_4_. Next, 0.8 mL of extraction solvent (containing 25 mg/L 2-methylpentanoic acid [internal standard] in methyl tert-butyl ether) was added. The mixture was vortexed for 10 s, shaken for 10 min, and sonicated in an ice-water bath for 10 min. Following centrifugation (4 °C, 10,000 rpm, 15 min), the sample was incubated at −20 °C for 30 min. Finally, the supernatant was transferred to an injection vial for GC-MS analysis following the standard procedures provided by Bio-Tree Biomedical Technology Co., Ltd. (Shanghai, China).

Analysis was conducted using a SHIMADZU GC2030-QP2020 NX gas chromatography-mass spectrometer (Shimadzu Corporation, Kyoto, Japan) equipped with an HP-FFAP capillary column (30 m × 250 μm × 0.25 μm, J&W Scientific, Folsom, CA, USA). A 1 μL aliquot of the sample was injected in split mode (5:1). High-purity helium served as the carrier gas with a constant flow rate of 1.2 mL min^−1^ through the column, and the front inlet purge flow was set at 3 mL min^−1^. The oven temperature program was initiated at 75 °C (held for 0 min), increased to 100 °C at 5 °C min^−1^ (held for 0 min), and subsequently raised to 240 °C at 30 °C min^−1^ with a final hold time of 5 min. The temperatures of the injection port, transfer line, and ion source were maintained at 240 °C, while the quadrupole was set at 150 °C. Electron impact ionization was employed at 70 eV. Mass spectrometric data were acquired in Scan/SIM mode, scanning from 33 to 150 *m*/*z* after a solvent delay of 2.90 min.

### 2.9. Statistical Analysis

All data are presented as mean ± standard deviation ($\overset{¯}{x}$ ± SD). Statistical analyses were performed using GraphPad Prism software (version 10.1.2; GraphPad Software, San Diego, CA, USA). For normally distributed data in multiple comparisons, one-way analysis of variance (ANOVA) was employed. followed by Dunnett’s multiple comparisons test; for heterogeneous variances, the Brown-Forsythe ANOVA test was employed. A threshold of * *p* < 0.05 was considered statistically significant.

## 3. Results

### 3.1. Propolis Effect on Body Weight Gain in HFD Rats

**Figure 1:**
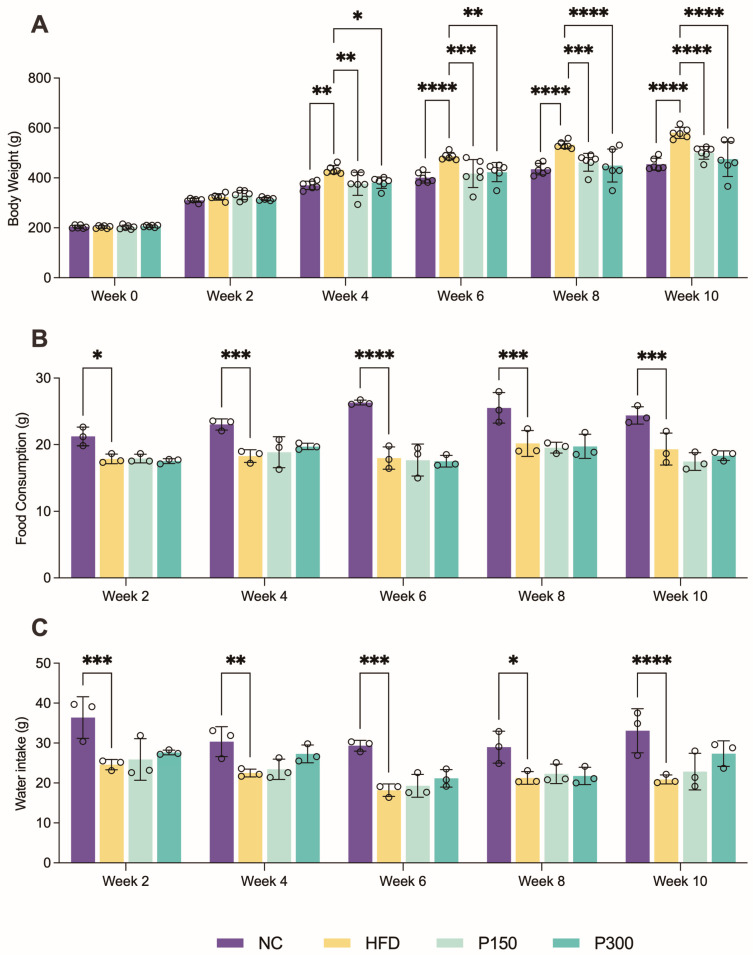
Effect of propolis on body weight (**A**), food consumption (**B**) and water intake (**C**) in HFD rats during administration. * *p* < 0.05; ** *p* < 0.01; *** *p* < 0.001; **** *p* < 0.0001 vs. HFD group (by one-way ANOVA with Dunnett’s post hoc test). NC: normal control group; HFD: high-fat diet rat group; P150: high-fat diet rats fed with 150 mg/kg propolis; P300: high-fat diet rats fed with 300 mg/kg propolis.

Rats in the HFD group exhibited a progressive increase in body weight, with statistically significant elevation compared to other groups from week 4 onward (*p* < 0.01), confirming successful establishment of the obesity model, as illustrated in Figure 1A. Notably, propolis intervention dose-dependently suppressed this weight gain, with rats in both P150 and P300 groups showing significantly lower body weight than that in the HFD group as early as week 4 (*p* < 0.05). By the experimental endpoint (week 10), the high dose propolis group (P300) demonstrated the most pronounced anti-obesity effect in body weight measurements.

Remarkably, this weight-reducing effect occurred independently of food intake modulation, as evidenced by comparable daily food consumption among HFD, P150, and P300 groups throughout the 10-week study period (*p* > 0.05, Figure 1B). The water intake analysis revealed that the NC group consumed significantly more water than the HFD group (*p* < 0.01), while propolis-treated groups showed a non-significant increase in water intake (P150 and P300 vs. HFD, *p* > 0.05, Figure 1C).

### 3.2. Propolis and Hepatic Steatosis in HFD Rats

**Figure 2:**
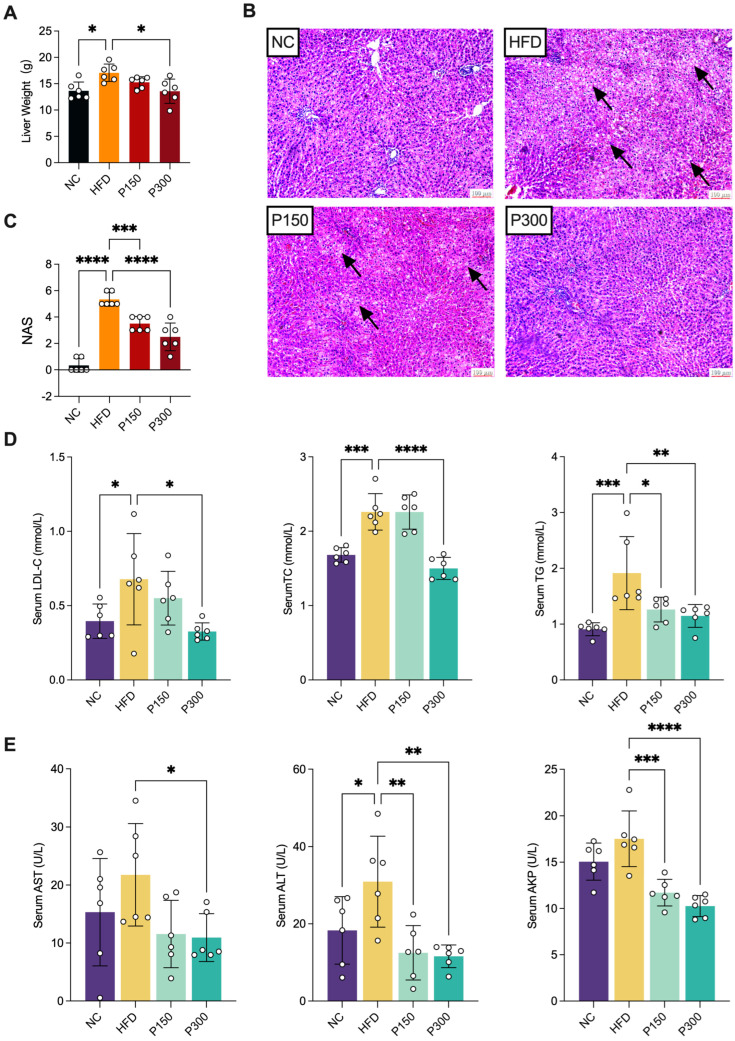
Effect of propolis on liver weight (**A**); liver histopathological structure (**B**), and the quantified histopathological score via nonalcoholic fatty liver disease activity score (NAS) (**C**); serum lipid indexes including LDL-c, TC, TG (**D**); serum liver indexes including AST, ALT, AKP (**E**) in HFD rats. * *p* < 0.05; ** *p* < 0.01; *** *p* < 0.001; **** *p* < 0.0001 vs. HFD group (by one-way ANOVA with Dunnett’s post hoc test). Representative histopathological images of liver sections stained with H&E, black arrows marked the cytoplasmic lipid vacuoles. The sections show the liver structure of rats in the NC (normal control), HFD (high-fat diet), P150 (propolis 150 mg/kg), and P300 (propolis 300 mg/kg) treatment groups.

Gravimetric analysis of liver tissues revealed a significant increase in liver weight in HFD-fed rats compared to NCs (*p* < 0.05, Figure 2A), indicating substantial lipid accumulation induced by the high-fat diet. Propolis intervention attenuated this effect in a dose-dependent manner, with the P150 group showing a moderate reduction and the P300 group exhibiting a statistically significant decrease in liver weight compared to HFD controls (*p* < 0.05, Figure 2A). Histopathological examinations via H&E staining (Figure 2B) revealed severe hepatic steatosis, inflammation, and ballooning in the HFD group, characterized by numerous cytoplasmic lipid vacuoles. Propolis treatment markedly ameliorated these pathological changes. As shown in Figure 2C, we observed a significantly higher NAS in the HFD group than in the NC group (*p* < 0.0001). Both propolis treatments significantly reduced the NAS in a dose-dependent manner (P150: *p* < 0.001; P300: *p* < 0.0001, vs. HFD group). Notably, the hepatic architecture in the P300 group was restored to a state close to normality. These quantitative findings demonstrate that propolis dose-dependently reverses HFD-induced liver injury.

As shown in Figure 2D, serum lipid analysis revealed that compared to the NC group, the HFD group exhibited significantly elevated levels of LDL-C, TC, and TG. Notably, high dose propolis treatment (P300) significantly reduced these serum lipid parameters compared to the HFD group. Additionally, serum liver function biomarkers demonstrated similar propolis’ protective effects against HFD-induced hepatic dysfunction (Figure 2E). The HFD group showed increased levels of AST, ALT, and AKP. In comparison to the HFD group, P300 group showed a 50% reduction in AST activity (*p* < 0.05), while the ALT enzyme activity in both P150 and P300 groups was significantly lower than that in the HFD group by 50% (*p* < 0.01), and the AKP enzyme activity decreased by 25% (*p* < 0.0001).

### 3.3. Propolis and Liver Lipid Metabolism in Rats

**Figure 3:**
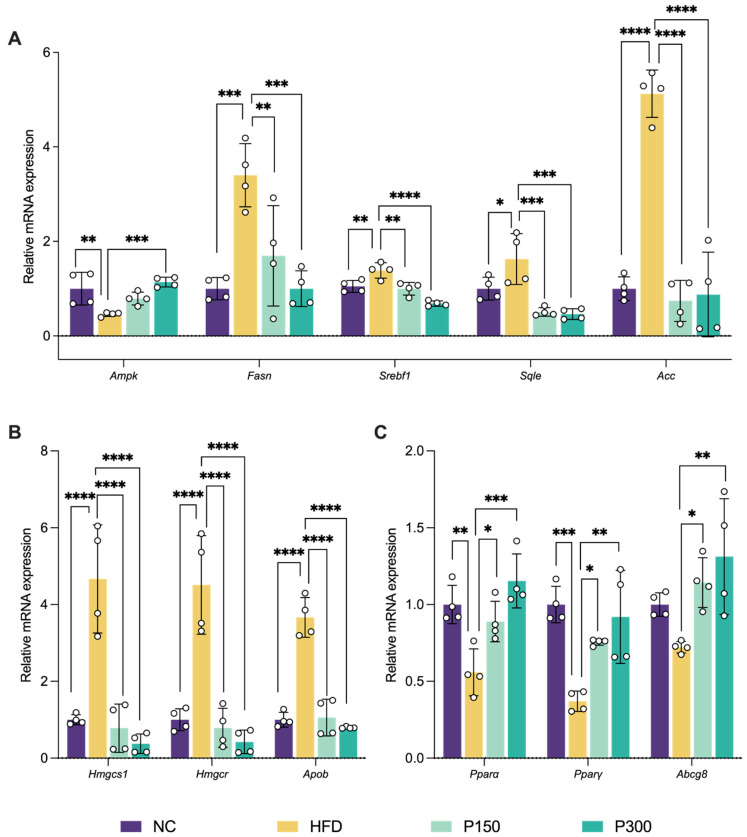
Effect of propolis on relative mRNA expression of hepatic fatty acids metabolism genes (**A**), hepatic cholesterol metabolism genes (**B**), and hepatic energy metabolism genes (**C**) in HFD rats. * *p* < 0.05; ** *p* < 0.01; *** *p* < 0.001; **** *p* < 0.0001, vs. HFD group (by one-way ANOVA with Dunnett’s post hoc test). NC: normal control group; HFD: high-fat diet rat group; P150: high-fat diet rats fed with 150 mg/kg propolis; P300: high-fat diet rats fed with 300 mg/kg propolis.

Propolis inhibits both cholesterol synthesis (via 3-hydroxy-3-methylglutaryl-CoA reductase (HMGCR, encoded by the gene *Hmgcr*) suppression) and fatty acid production (via downregulation of *Srebf1*/*Fasn*/*Acaca*) by activating the AMPK signaling pathway. As shown in Figure 3A, high-fat diet (HFD) significantly suppressed hepatic *Ampk* expression (*p* < 0.01) while upregulating key fatty acid synthesis genes, including *Srebf1*, *Fasn*, *Acaca*, and squalene epoxidase (*Sqle*) (*p* < 0.05–0.0001). Notably, high dose propolis intervention (P300) not only restored *Ampk* expression (*p* < 0.001) but also significantly inhibited the overexpression of these lipogenic genes (*p* < 0.01). Regarding cholesterol metabolism, as shown in Figure 3B, HFD feeding markedly upregulated the mRNA expression of rate-limiting enzymes 3-hydroxy-3-methylglutaryl-CoA synthase 1 (*Hmgcs1*) and *Hmgcr*, along with apolipoprotein B (*Apob*) (*p* < 0.0001). Propolis treatment effectively reversed these pathological alterations (*p* < 0.0001). Further investigation revealed that HFD reduced the expression of both *Pparα* and *Pparγ* (*p* < 0.01 and *p* < 0.001, respectively, Figure 3C), as well as the expression of ATP binding cassette subfamily G member 8 (*Abcg8*) (*p* > 0.05), while propolis administration restored their expression to near-normal levels. These results indicate that propolis promotes fatty acid β-oxidation through upregulation of *Ppars* and *Abcg8*. This effect is consistent with the previously observed modulation of cholesterol and fatty acid metabolism, as both processes are critically mediated via the AMPK signaling pathway, underscoring the integral role of AMPK in lipid metabolic regulation.

### 3.4. Propolis Effect on Colonic Structure and Enteroendocrine Hormone Secretion in HFD Rats

**Figure 4:**
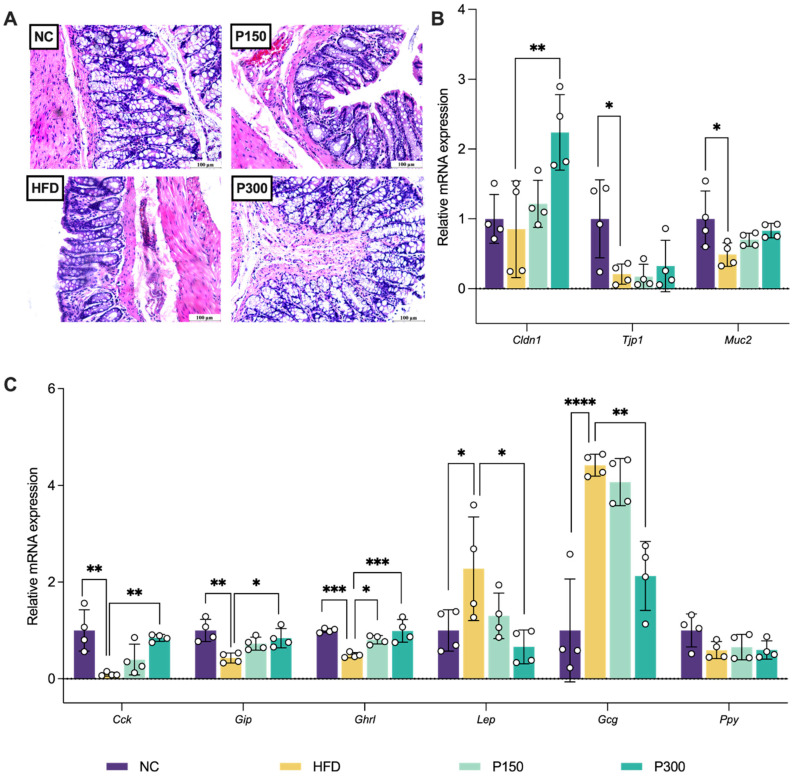
Effect of propolis on gut histopathological structure of different groups from H&E staining (×100, (**A**)), relative mRNA expression of tight junction proteins in the gut (**B**), and gut hormone related genes (**C**) in HFD rats. * *p* < 0.05; ** *p* < 0.01; *** *p* < 0.001; **** *p* < 0.0001 vs. HFD group (by one-way ANOVA with Dunnett’s post hoc test). NC: normal control group; HFD: high-fat diet rat group; P150: high-fat diet rats fed with 150 mg/kg propolis; P300: high-fat diet rats fed with 300 mg/kg propolis.

In the gut, the homeostasis of the gut barrier enables the normal development of various metabolic reactions in the body, among which intestinal tight junction proteins play a key role [34,35]. Propolis preserved intestinal structural integrity through histopathological improvement and tight junction protein regulation. Histopathological analysis revealed distinct morphological variations in colonic tissues among the examined groups (Figure 4A). The NC group maintained intact mucosal architecture with regularly arranged epithelial cells, whereas the HFD group exhibited significant mucosal edema in the muscular layer. Propolis treatment dose-dependently improved these structural damages, with the P150 showing partial resolution of edema. The architecture of the colonic mucosa in the P300 group showed a notable restoration, closely resembling that of the NC group. At the molecular level (Figure 4B), HFD feeding significantly compromised the intestinal barrier function, as evidenced by marked downregulation of tight junction protein 1 (*Tjp1*) (*p* < 0.05 vs. NC), reduced mucin 2 (*Muc2*) expression (*p* < 0.05), and unaltered Claudin-1 (encoded by the gene *Cldn1*) levels (*p* > 0.05). Propolis treatment, particularly at high dose (P300), exhibited promising restorative effects including, an upward trend in *Tjp1* and *Muc2* expression, and significant recovery of *Cldn1* to baseline levels (*p* < 0.01 vs. HFD). These findings suggest that propolis reinforces intestinal barrier integrity through Cldn1-mediated tight junction reconstruction.

Furthermore, propolis’ remarkable ability to modulate enteroendocrine hormone secretion was demonstrated (Figure 4C). Compared to NC group, HFD feeding significantly suppressed the expression of *Cck* (*p* < 0.01), *GIP* (*p* < 0.01), and *Ghrl* (*p* < 0.001). Propolis treatment dose-dependently restored these hormonal profiles, i.e., propolis at 150 mg/kg significantly upregulated *Ghrl* (*p* < 0.05), while propolis at 300 mg/kg completely normalized the mRNA expression levels of *Cck* (*p* < 0.01 vs. HFD), *Gip* (*p* < 0.05 vs. HFD), *Ghrl* (*p* < 0.001 vs. HFD), and *Lep* (*p* < 0.05 vs. HFD). Additionally, we observed HFD-induced hyperexpression of GCG (*p* < 0.001) and dose-dependent GCG suppression by propolis.

### 3.5. Effects of Propolis on Gut Microbiota Diversity in HFD Rats

**Figure 5:**
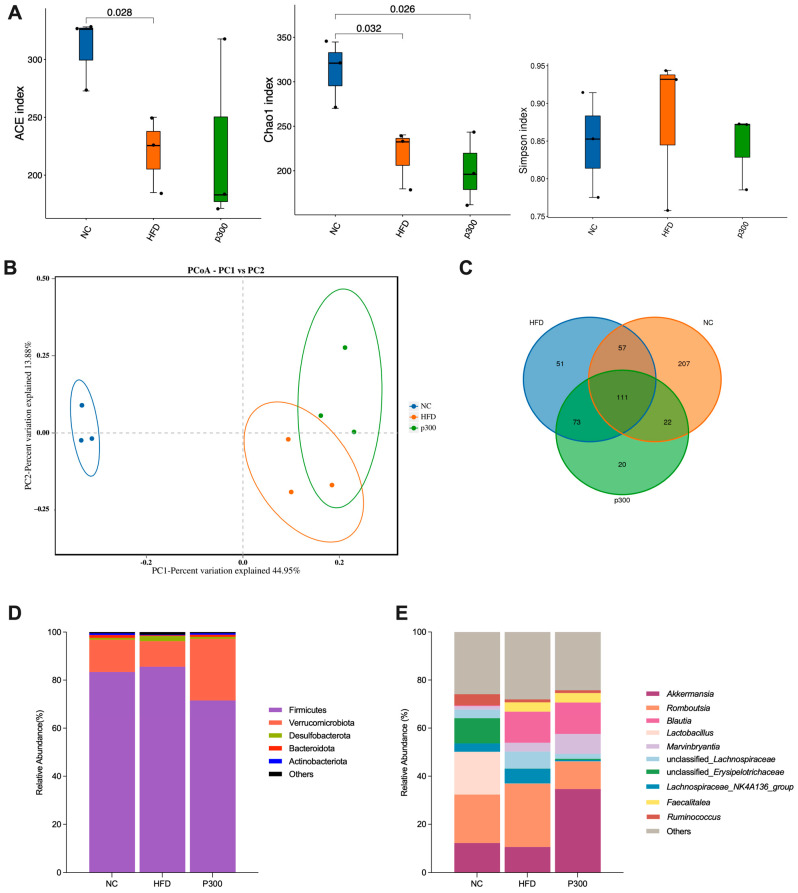
Effects of propolis on gut microbiota diversity analyzed by alpha diversity analysis (**A**), beta diversity analysis (**B**), and feature Venn diagram (**C**), and differences in the composition of gut microbiota at the phylum (**D**), and genus (**E**) levels. NC: normal control group; HFD: high-fat diet rat group; P300: high-fat diet rats fed with 300 mg/kg propolis.

In the Alpha diversity analysis, using the ACE index and Chao1 index, it was found that a high-fat diet reduced species richness in the population, no significant recovery was observed in the P300 group (Figure 5A). The Simpson index, which measures species diversity, indicates lower diversity with higher values. According to the Simpson index, species diversity in the HFD group was lower than in the NC and P300 groups. In the PCoA plot based on Beta diversity analysis, significant differences were observed among the microbiota of the NC, HFD, and P300 groups (Figure 5B). The Venn diagram in Figure 5C highlights the shared and unique OTUs among different groups. Specifically, the NC group has 207 unique OTUs, the HFD group has 51 unique OTUs, and the P300 group has 20 unique OTUs (Figure 5C).

At the phylum level, the top five dominant phyla among the three groups of gut microbiota, ranked by relative abundance, were Firmicutes, Verrucomicrobia, Desulfobacterota, Bacteroidetes, and Actinobacteria (Figure 5D). Notably, treatment with propolis reduced the relative abundance of the Firmicutes phylum (by approximately 12%) and increased that of Verrucomicrobia (by about 18%) and Bacteroidota. At the genus level, observations indicate that the use of propolis modulates the microbial fluctuations induced by a high-fat diet. For instance, propolis increased the relative abundance of *Akkermansia* while decreasing that of *Romboutsia* and *Lachnospiraceae*, thereby normalizing the microbial composition. Interestingly, propolis did not restore the reduction in *Lactobacillus* caused by HFD, suggesting a selective stimulation effect of propolis on different probiotics (Figure 5E).

### 3.6. Propolis Effect on Gut Microbiota Composition and SCFAs in HFD Rats

**Figure 6:**
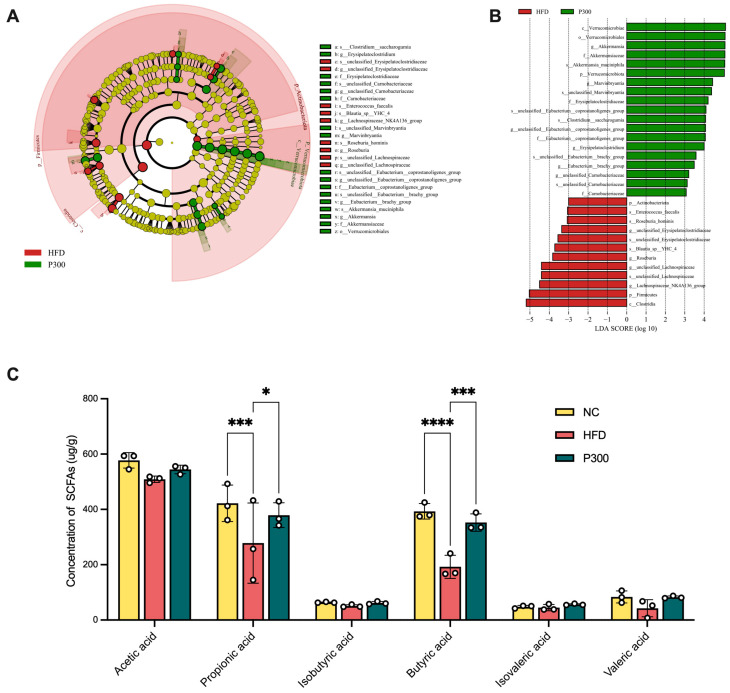
LEfSe analysis of gut microbiota represented by evolutionary branching plots (**A**), and histogram of LDA value distribution (LDA > 3, (**B**)), comparing the HFD and propolis-treated (P300) groups. Effect of propolis on colonic short-chain fatty acids in the NC, HFD, and propolis-treated (P300) groups (**C**). * *p* < 0.05; *** *p* < 0.001; **** *p* < 0.0001 vs. HFD group (by one-way ANOVA with Dunnett’s post hoc test). NC: normal control group; HFD: high-fat diet rat group; P300: high-fat diet rats fed with 300 mg/kg propolis.

The differences in gut microbiota between the propolis treatment group and the high-fat diet group were noted. The differential gut microbiota of HFD and P300 groups were analyzed using LefSe (Figure 6A) and LDA (Figure 6B, with an LDA effect size > 3 and *p* < 0.05). After gavage with a high dose of propolis, the gut microbiota of rats in the P300 group changed, with *Verrucomicrobia* being the dominant phylum at the phylum level, and *Akkermansia*, *Marvinbryantia*, unclassified *Eubacterium coprostanoligenes* group, *Erysipelatoclostridium*, *Eubacterium Brachy* group, unclassified *Carnobacteriaceae* being dominant genera at the genus level. Considering that the advantages of these bacterial communities are recognized as SCFAs-producing bacteria, it was important to focus on whether propolis consistently improves SCFAs. SCFAs are produced by the metabolic breakdown of specific colonic anaerobic bacteria, with acetate and propionate playing positive roles in gut metabolism and being involved in lipid metabolism regulation. As shown in Figure 6C, all SCFAs in the HFD group were lower than those in the NC group, with a significant reduction in propionic acid (*p* < 0.001) and butyric acid (*p* < 0.0001). It was interesting that under the influence of propolis, SCFAs levels were recovered to some extent, especially with a significant increase in propionic acid (*p* < 0.05) and butyric acid (*p* < 0.001) content.

### 3.7. The Dominant Bacteria in the Regulation of Gut Hormones and Lipid Metabolism by Propolis

**Figure 7:**
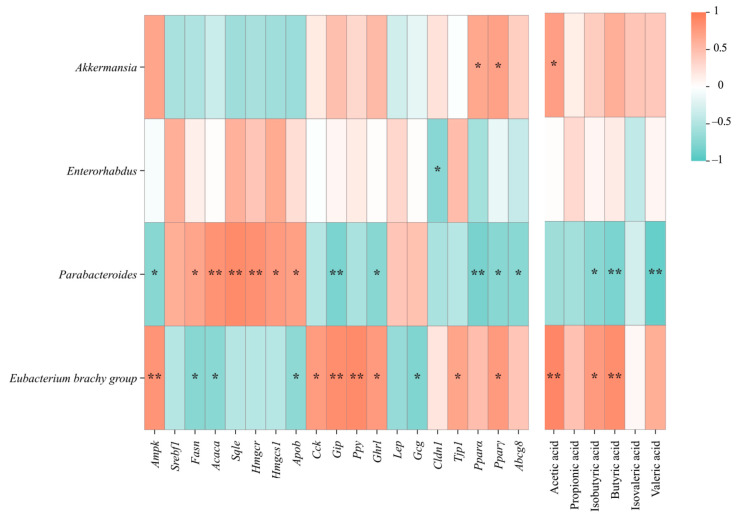
Correlation analysis of gut microbiota with metabolic genes and SCFAs via the Spearman rank correlation. * *p* < 0.05; ** *p* < 0.01.

The correlation of the whole microflora at the genus level was analyzed by the Spearman rank correlation coefficient, combined with the results of the differential microflora, and other microflora with high correlation levels were screened out (* *p* < 0.05, ** *p* < 0.01), and the correlation heat map was constructed. As shown in Figure 7, the association between the dominant bacteria and the genes related to liver lipid metabolism was first analyzed. Among the genes of liver fatty acid synthesis and cholesterol metabolism, *Eubacterium Brachy* group was significantly positively correlated with the mRNA expression of *Ampk*, and negatively correlated with *Fasn*, *Acaca*, and *Apob*. There was a significant negative correlation between *Parabacteroides* and the *Ampk* expression level, while *Parabacteroides* were significantly positively correlated with *Fasn*, *Acaca*, *Sqle*, *Hmgcr*, *Hmgcs1*, and *Apob*. Then, the microbiota related to intestinal hormones were analyzed and it was found that *Eubacterium Brachy* group was significantly positively correlated with *Cck*, *Gip*, pancreatic polypeptide (*Ppy*), *Ghrl*, and negatively correlated with *Lep* and *Gcg*. In addition, a significant negative correlation with *Gip* and *Ghrl*, *Parabacteroides* showed no significant association with other gut hormones. It was also found that *Eubacterium brachy* group was significantly positively correlated with *Tjp1*, and *Enterococcus* spp. was negatively correlated with *Cldn1*. In the gut, some bacteria can produce SCFAs, and these metabolites can regulate changes in intestinal hormone secretion. The *Eubacterium brachy* group was found to be significantly positively correlated with acetic acid, isobutyric acid, and butyric acid. Furthermore, *Akkermansia* was significantly positively correlated with acetic acid, along with a significant negative correlation between *Parabacteroides* and isobutyric acid, butyric acid and valeric acid.

## 4. Discussion

The present study demonstrates that propolis confers protection against HFD-induced metabolic dysfunction through multi-targeted modulation of gut–liver signaling pathways. A key and novel finding is that propolis reduced weight gain and improved metabolic parameters without suppressing appetite (Figure 1), suggesting mechanisms beyond mere caloric restriction. This result was largely consistent with the existing literature regarding the presence of polyphenols, such as quercetin [36] and caffeic acid [37] in propolis. This insight refines our understanding of propolis’s anti-obesity action and underscores the importance of the gut–liver crosstalk.

Our data strongly implicate the AMPK pathway as a central mediator of the improved hepatic lipid metabolism observed with propolis treatment. It was found that decreased *Ampk* expression in the HFD group was accompanied by abnormal upregulation of genes involved in fatty acid synthesis (*Srebf1*, *Fasn*, *Acaca*, *Apob*, *Sqle*), cholesterol synthesis (*Hmgcs1*, *Hmgcr*), and the reduction in fatty acid *β*-oxidation related genes (*Pparα*, *Pparγ*). This transcriptional reprogramming is consistent with AMPK’s established role as a cellular energy sensor that inhibits anabolic and promotes catabolic pathways [38,39]. Additionally, propolis markedly upregulated *Pparα*, *Pparγ*, and *Abcg8* orchestrating multi-target metabolic regulation. PPARα induces carnitine palmitoyltransferase 1 to accelerate mitochondrial fatty acid catabolism, while PPARγ enhances adipocyte differentiation, redirecting lipids to adipose tissue and preventing ectopic deposition [40,41]. Studies have shown that propolis regulates lipid metabolism in obese rats by upregulating the expression of PPARα and PPARγ, which was consistent with the results [42,43]. ABCG8 plays a key role in lipid homeostasis by facilitating reverse cholesterol transport and promoting biliary cholesterol excretion, thereby reducing the efficiency of intestinal cholesterol absorption [44]. Numerous studies have highlighted the regulatory effects of polyphenol-rich natural products on ABCG8, suggesting that these compounds can ameliorate lipid metabolism through modulation of this transporter [45,46,47,48]. Our findings are consistent with this mechanism, demonstrating that propolis—a naturally polyphenol-rich substance—similarly upregulates ABCG8 expression, thereby contributing to its lipid-modulating benefits. In conclusion, the results strengthen the concept that propolis improves hepatic lipid metabolism through the AMPK signaling pathways.

Addressing the initial observation of efficacy without anorexia, we investigated gut hormones and found that propolis orchestrated beneficial reprogramming. Gut hormones could regulate lipid accumulation in the body by influencing eating behavior or affecting peripheral signals [49]. Propolis restored *Cck*/*Gip* (insulinotropic hormones) and *Ghrl* (feeding regulator) levels, achieving dual modulation of “peripheral glucose utilization” and “central feeding behavior” [50,51]. Elevated *Lep* in the HFD group reflected obesity-associated leptin resistance, which propolis significantly alleviated to restore leptin receptor sensitivity. This modulation of gut hormones represents a particularly underappreciated aspect of propolis’s mechanism and could contribute to improved glucose homeostasis and restored leptin sensitivity, potentially via indirect crosstalk with AMPK signaling [52,53].

The secretion of gut hormones is closely related to gut homeostasis [11].Therefore, the stability of the intestinal microenvironment, which includes the tight junctions of the gut and the metabolism of the gut microbiota, was further observed. In terms of the gut barrier, HFD induced significant pathological changes in the colon, such as mucosal edema and downregulation of the mRNA expression levels of essential tight junction proteins (*Tjp1* and *Cldn-1*), all of which were effectively mitigated by propolis intervention. This may be related to the upregulation of *Ppars* expression previously observed, with the view that *Pparγ* could promote the transcription of tight junction protein genes [54,55]. Muc2, secreted by goblet cells, serves as a protective mucus in the gastrointestinal tract. Acting as a primary component of the intestinal mucus layer, it aids in forming a physical barrier that shields the intestinal epithelial cells from pathogens, toxins, and mechanical damage [40]. The findings indicated that propolis treatment restored intestinal barrier function and *Muc2* expression.

In terms of gut microbiota, our study provides a novel insight into the interaction between propolis and the gut microbiota, with significant translational implications. While our finding that propolis does not reverse HFD-induced reductions in alpha diversity aligns with previous reports [20,56], it induced a selective enrichment of specific beneficial taxa, including short-chain fatty acid (SCFA)-producers like *Akkermansia*, *Roseburia*, and the *Eubacterium brachy* group instead. This suggests that the therapeutic potential of propolis may lie in its ability to precision-target key functional players rather than broadly resetting the entire microbial community, a strategy that could be more feasible in clinical settings.

At the phylum level, propolis treatment significantly increased the relative abundance of *Verrucomicrobia* (*p* < 0.01), a mucin-dwelling phylum known to degrade complex polysaccharides (mucins and cellulose), provide essential nutrients and energy [57], and maintain intestinal mucosal health. Notably, at the genus level, the unclassified *Erysipelatoclostridiaceae*, a bacterial genus belonging to the *Firmicutes phylum*, is involved in carbohydrate metabolism and showed a relationship between SCFAs [58]. *Marvinbryantia* and *Roseburia* can metabolize to produce butyric acid, which participates in regulating metabolic disorders induced by a high-fat diet and promotes the establishment of a stable intestinal environment [59,60,61], and the genus *Akkermansia* has long been established as one of the key beneficial bacteria closely associated with obesity management [62]. Intestinal beneficial bacteria maintain gut homeostasis by increasing the production of SCFAs through metabolism and enhancing the thickness of the intestinal mucosa. Butyric acid and propionic acid are typically metabolic byproducts of probiotics, and they can reduce body weight and decrease HFD-induced hepatic steatosis [63]. Propolis increased the levels of butyric acid and propionic acid, with reports indicating that *Bacteroides*, *Roseburia*, and *Ruminococcus*, which are butyric acid-producing genera, generate propionate through pathways involving succinate, propionate esters, and propanediol [64].

Correlation analysis found that the dominant bacteria *Eubacterium brachy* group, a known butyrate producer [65], as a potential microbial hub, showing significant associations with SCFA levels, gut hormone expression, and AMPK pathway activity. Research has found that butyrate can regulate satiety hormones such as ghrelin and GLP-1 thereby influencing obesity or diabetes [66]. The *Eubacterium* genus regulates intestinal barrier function and gut hormones by modulating butyrate metabolism, which aligns with our findings.

While our study provides compelling evidence for the involvement of the gut hormone axis and AMPK signaling in the anti-obesity effects of propolis, we acknowledge its limitations. First, it should be noted that a detailed chemical fingerprint of the propolis was not performed. Future studies would benefit from such analysis to account for compositional variability and to identify the key bioactive compounds responsible for the effects on the gut–liver axis. Second, while these mRNA-level findings are consistent with AMPK’s established role as a cellular energy sensor, we acknowledge that future validation at the protein level is necessary to fully confirm pathway activation. Third, the sample size for 16S rRNA sequencing and SCFA analysis (*n* = 3 per group), while suitable to reveal large-effect-size changes consistent with previous studies in the field [67,68], may limit the statistical power to detect more subtle microbial shifts. Future studies with larger cohort sizes will be valuable to validate these associations and explore more nuanced microbiota-host interactions. Additionally, although our integrated multi-omics approach reveals compelling associations, the exact causal relationships within the proposed gut microbiota–gut hormone–liver AMPK axis remain to be fully elucidated. Specifically, it is unclear whether AMPK activation is a direct target of propolis or a secondary effect of microbiota remodeling, and the pivotal role of the *Eubacterium brachy* group identified by correlation analysis requires functional validation through future interventional studies, such as the use of AMPK inhibitors or bacterial colonization models. Despite these limitations, the translational relevance of our findings is underscored by the clinical feasibility of the propolis doses used. Based on established dose conversion factors [69], the administered doses in this study (150 and 300 mg/kg in rats) correspond to approximately 1.7 g and 3.4 g, respectively, for a 70 kg adult. The higher dose (3.4 g) is comparable to the daily recommendation of many commercial propolis supplements, highlighting the potential practical applicability of our results.

## 5. Conclusions

In conclusion, our study demonstrates that propolis ameliorates HFD-induced metabolic disorders by orchestrating a multi-targeted interplay among the gut microbiota, enteroendocrine hormones, and the hepatic AMPK pathway. The identification of gut hormone modulation as a key mechanism, independent of appetite suppression, provides a novel perspective on propolis’s anti-obesity effects. While this work highlights the multi-target nature of propolis, future studies are warranted to delineate the causal relationships within this axis and to identify the specific bioactive compounds responsible for these benefits. Nonetheless, our findings solidify the scientific basis for using propolis as a functional food or natural supplement for managing lipid metabolism disorders.

## Data Availability

The raw data supporting the conclusions of this article will be made available by the authors on request due to legal and ethical restrictions concerning confidentiality and personal data protection.

## Supplementary Materials

The following supporting information can be downloaded at: https://www.mdpi.com/article/10.3390/nu17193114/s1, Table S1: Primer Sequences.

## References

[1] Stewart J., McCallin T., Martinez J., Chacko S., Yusuf S. (2020). Hyperlipidemia. Pediatr. Rev..

[2] Bergmann N.C., Davies M.J., Lingvay I., Knop F.K. (2023). Semaglutide for the treatment of overweight and obesity: A review. Diabetes Obes. Metab..

[3] Gribble F.M., Reimann F. (2019). Function and mechanisms of enteroendocrine cells and gut hormones in metabolism. Nat. Rev. Endocrinol..

[4] Madison B.B. (2016). Srebp2: A master regulator of sterol and fatty acid synthesis. J. Lipid Res..

[5] Eberle D., Hegarty B., Bossard P., Ferre P., Foufelle F. (2004). SREBP transcription factors: Master regulators of lipid homeostasis. Biochimie.

[6] Fang C., Pan J., Qu N., Lei Y., Han J., Zhang J., Han D. (2022). The AMPK pathway in fatty liver disease. Front. Physiol..

[7] Janovska A., Hatzinikolas G., Staikopoulos V., McInerney J., Mano M., Wittert G.A. (2008). AMPK and ACC phosphorylation: Effect of leptin, muscle fibre type and obesity. Mol. Cell. Endocrinol..

[8] Peng I.C., Chen Z., Sun W., Li Y.S., Marin T.L., Hsu P.H., Su M.I., Cui X., Pan S., Lytle C.Y. (2012). Glucagon regulates ACC activity in adipocytes through the CAMKKbeta/AMPK pathway. Am. J. Physiol. Endocrinol. Metab..

[9] Kim S.J., Nian C., McIntosh C.H. (2007). Activation of lipoprotein lipase by glucose-dependent insulinotropic polypeptide in adipocytes. A role for a protein kinase B, LKB1, and AMP-activated protein kinase cascade. J. Biol. Chem..

[10] Mukherjee A., Lordan C., Ross R.P., Cotter P.D. (2020). Gut microbes from the phylogenetically diverse genus *Eubacterium* and their various contributions to gut health. Gut Microbes.

[11] Zhao X., Qiu Y., Liang L., Fu X. (2025). Interkingdom signaling between gastrointestinal hormones and the gut microbiome. Gut Microbes.

[12] Shimizu H., Masujima Y., Ushiroda C., Mizushima R., Taira S., Ohue-Kitano R., Kimura I. (2019). Dietary short-chain fatty acid intake improves the hepatic metabolic condition via FFAR3. Sci. Rep..

[13] Joyce S.A., MacSharry J., Casey P.G., Kinsella M., Murphy E.F., Shanahan F., Hill C., Gahan C.G. (2014). Regulation of host weight gain and lipid metabolism by bacterial bile acid modification in the gut. Proc. Natl. Acad. Sci. USA.

[14] Altabbal S., Athamnah K., Rahma A., Wali A.F., Eid A.H., Iratni R., Al Dhaheri Y. (2023). Propolis: A Detailed Insight of Its Anticancer Molecular Mechanisms. Pharmaceuticals.

[15] Salehi-Sahlabadi A., Chhabra M., Rahmani J., Momeni A., Karam G., Nattagh-Eshtivani E., Nouri M., Clark C., Salehi P., Hekmatdoost A. (2020). The effect of propolis on anthropometric indices and lipid profile: A systematic review and meta-analysis of randomized controlled trials. J. Diabetes Metab. Disord..

[16] Soleimani D., Miryan M., Tutunchi H., Navashenaq J.G., Sadeghi E., Ghayour-Mobarhan M., Ferns G.A., Ostadrahimi A. (2021). A systematic review of preclinical studies on the efficacy of propolis for the treatment of inflammatory bowel disease. Phytother. Res..

[17] Koya-Miyata S., Arai N., Mizote A., Taniguchi Y., Ushio S., Iwaki K., Fukuda S. (2009). Propolis prevents diet-induced hyperlipidemia and mitigates weight gain in diet-induced obesity in mice. Biol. Pharm. Bull..

[18] Ichi I., Hori H., Takashima Y., Adachi N., Kataoka R., Okihara K., Hashimoto K., Kojo S. (2009). The beneficial effect of propolis on fat accumulation and lipid metabolism in rats fed a high-fat diet. J. Food Sci..

[19] Chien Y.H., Yu Y.H., Chen Y.W. (2023). Taiwanese green propolis ameliorates metabolic syndrome via remodeling of white adipose tissue and modulation of gut microbiota in diet-induced obese mice. Biomed. Pharmacother..

[20] Xue M., Liu Y., Xu H., Zhou Z., Ma Y., Sun T., Liu M., Zhang H., Liang H. (2019). Propolis modulates the gut microbiota and improves the intestinal mucosal barrier function in diabetic rats. Biomed. Pharmacother..

[21] Wang K., Jin X.L., Shen X.G., Sun L.P., Wu L.M., Wei J.Q., Marcucci M.C., Hu F.L., Liu J.X. (2016). Effects of Chinese Propolis in Protecting Bovine Mammary Epithelial Cells against Mastitis Pathogens-Induced Cell Damage. Mediators Inflamm..

[22] Song W.Y., Aihara Y., Hashimoto T., Kanazawa K., Mizuno M. (2015). (-)-Epigallocatechin-3-gallate induces secretion of anorexigenic gut hormones. J. Clin. Biochem. Nutr..

[23] de Melo T.S., Lima P.R., Carvalho K.M., Fontenele T.M., Solon F.R., Tome A.R., de Lemos T.L., da Cruz Fonseca S.G., Santos F.A., Rao V.S. (2017). Ferulic acid lowers body weight and visceral fat accumulation via modulation of enzymatic, hormonal and inflammatory changes in a mouse model of high-fat diet-induced obesity. Braz. J. Med. Biol. Res..

[24] Goncalves V.C., Pinheiro D., de la Rosa T., de Almeida A.G., Scorza F.A., Scorza C.A. (2020). Propolis as A Potential Disease-Modifying Strategy in Parkinson’s Disease: Cardioprotective and Neuroprotective Effects in the 6-OHDA Rat Model. Nutrients.

[25] Zheng Y., Wu Y., Tao L., Chen X., Jones T.J., Wang K., Hu F. (2020). Chinese Propolis Prevents Obesity and Metabolism Syndromes Induced by a High Fat Diet and Accompanied by an Altered Gut Microbiota Structure in Mice. Nutrients.

[26] Sun Y., Han M., Shen Z., Huang H., Miao X. (2018). Anti-hypertensive and cardioprotective effects of a novel apitherapy formulation via upregulation of peroxisome proliferator-activated receptor-alpha and -gamma in spontaneous hypertensive rats. Saudi J. Biol. Sci..

[27] Song Y., Zhang Y., Di Y., Li N., Zhao Z., Liu Z., Sun L., Liu X., Wang Y. (2025). Chicoric Acid Differentially Ameliorates Circadian Rhythm Disorder-Induced Liver Glucose Homeostasis Dysregulation in Mice Depending on Intervention Time. J. Agric. Food Chem..

[28] Liu Y., Yang K., Jia Y., Shi J., Tong Z., Fang D., Yang B., Su C., Li R., Xiao X. (2021). Gut microbiome alterations in high-fat-diet-fed mice are associated with antibiotic tolerance. Nat. Microbiol..

[29] Kelly B.J., Gross R., Bittinger K., Sherrill-Mix S., Lewis J.D., Collman R.G., Bushman F.D., Li H. (2015). Power and sample-size estimation for microbiome studies using pairwise distances and PERMANOVA. Bioinformatics.

[30] Wang T., Huang Y., Hu M., Huang X., Yu D., Jia L., Zhi W., Mu Y., Zhou Z., Wang J. (2025). Saponins from Panax japonicus Enhance Lipolysis via Acting on FGF21-beta-Klotho/FGFR1 in Obese Mice. J. Agric. Food Chem..

[31] Kleiner D.E., Brunt E.M., Van Natta M., Behling C., Contos M.J., Cummings O.W., Ferrell L.D., Liu Y.C., Torbenson M.S., Unalp-Arida A. (2005). Design and validation of a histological scoring system for nonalcoholic fatty liver disease. Hepatology.

[32] Yang L., Liu R., Meng Y., Deng Z., Bu S., Liu J., Huang A., Wu S., Kan X. (2025). Fruit Phenotype Analysis of SlSAHH2-CRISPR Tomato and Methylation Mechanism of SlSAHH2 Promoting Fruit Ripening. J. Agric. Food Chem..

[33] Qiu K., Pan Y., Huang W., Li M., Yan X., Zhou Z., Qi J. (2023). CXCL5 Promotes Acetaminophen-Induced Hepatotoxicity by Activating Kupffer Cells. Int. J. Mol. Sci..

[34] Ma J., Piao X., Mahfuz S., Long S., Wang J. (2022). The interaction among gut microbes, the intestinal barrier and short chain fatty acids. Anim. Nutr..

[35] Neurath M.F., Artis D., Becker C. (2025). The intestinal barrier: A pivotal role in health, inflammation, and cancer. Lancet Gastroenterol. Hepatol..

[36] Rivera L., Moron R., Sanchez M., Zarzuelo A., Galisteo M. (2008). Quercetin ameliorates metabolic syndrome and improves the inflammatory status in obese Zucker rats. Obesity.

[37] Kim H.M., Kim Y., Lee E.S., Huh J.H., Chung C.H. (2018). Caffeic acid ameliorates hepatic steatosis and reduces ER stress in high fat diet-induced obese mice by regulating autophagy. Nutrition.

[38] Li Z., Huang Q., Zheng Y., Zhang Y., Liu B., Shi W., Zeng Z. (2023). Kaempferitrin: A Flavonoid Marker to Distinguish Camellia oleifera Honey. Nutrients.

[39] Li N., Yin L., Shang J., Liang M., Liu Z., Yang H., Qiang G., Du G., Yang X. (2023). Kaempferol attenuates nonalcoholic fatty liver disease in type 2 diabetic mice via the Sirt1/AMPK signaling pathway. Biomed. Pharmacother..

[40] Pelaseyed T., Bergstrom J.H., Gustafsson J.K., Ermund A., Birchenough G.M., Schutte A., van der Post S., Svensson F., Rodriguez-Pineiro A.M., Nystrom E.E. (2014). The mucus and mucins of the goblet cells and enterocytes provide the first defense line of the gastrointestinal tract and interact with the immune system. Immunol. Rev..

[41] Ahmadian M., Suh J.M., Hah N., Liddle C., Atkins A.R., Downes M., Evans R.M. (2013). PPARgamma signaling and metabolism: The good, the bad and the future. Nat. Med..

[42] Chen L.H., Chien Y.W., Chang M.L., Hou C.C., Chan C.H., Tang H.W., Huang H.Y. (2018). Taiwanese Green Propolis Ethanol Extract Delays the Progression of Type 2 Diabetes Mellitus in Rats Treated with Streptozotocin/High-Fat Diet. Nutrients.

[43] Kong L., Zhang Y., Feng Z., Dong J., Zhang H. (2021). Phenolic Compounds of Propolis Alleviate Lipid Metabolism Disorder. Evid. Based Complement. Alternat Med..

[44] Yu X.H., Qian K., Jiang N., Zheng X.L., Cayabyab F.S., Tang C.K. (2014). ABCG5/ABCG8 in cholesterol excretion and atherosclerosis. Clin. Chim. Acta.

[45] Liu S., You L., Zhao Y., Chang X. (2018). Wild Lonicera caerulea berry polyphenol extract reduces cholesterol accumulation and enhances antioxidant capacity in vitro and in vivo. Food Res. Int..

[46] Jeon S., Lee S., Choi Y., Kim B. (2021). The Effects of Polyphenol-Rich Black Elderberry on Oxidative Stress and Hepatic Cholesterol Metabolism. Appl. Sci..

[47] Ren J., Zhang X., Heiyan-Perhat S.U., Yang P., Han H., Li Y., Gao J., He E., Li Y. (2024). Therapeutic Role of Polyphenol Extract from Prunus cerasifera Ehrhart on Non-Alcoholic Fatty Liver. Plants.

[48] Li D., Cui Y., Wang X., Liu F., Li X. (2021). Apple Polyphenol Extract Improves High-Fat Diet-Induced Hepatic Steatosis by Regulating Bile Acid Synthesis and Gut Microbiota in C57BL/6 Male Mice. J. Agric. Food Chem..

[49] Murphy K.G., Bloom S.R. (2006). Gut hormones and the regulation of energy homeostasis. Nature.

[50] Bauer P.V., Hamr S.C., Duca F.A. (2016). Regulation of energy balance by a gut-brain axis and involvement of the gut microbiota. Cell. Mol. Life Sci..

[51] Irwin N., Montgomery I.A., O’Harte F.P., Frizelle P., Flatt P.R. (2013). Comparison of the independent and combined metabolic effects of subchronic modulation of CCK and GIP receptor action in obesity-related diabetes. Int. J. Obes..

[52] Jiang M., He J., Sun Y., Dong X., Yao J., Gu H., Liu L. (2021). Leptin Induced TLR4 Expression via the JAK2-STAT3 Pathway in Obesity-Related Osteoarthritis. Oxid. Med. Cell. Longev..

[53] Zhang B., Chen X., Xie C., Chen Z., Liu Y., Ru F., He Y. (2020). Leptin promotes epithelial-mesenchymal transition in benign prostatic hyperplasia through downregulation of BAMBI. Exp. Cell Res..

[54] Varley C.L., Garthwaite M.A., Cross W., Hinley J., Trejdosiewicz L.K., Southgate J. (2006). PPARgamma-regulated tight junction development during human urothelial cytodifferentiation. J. Cell. Physiol..

[55] Zhao J., Zhao R., Cheng L., Yang J., Zhu L. (2018). Peroxisome proliferator-activated receptor gamma activation promotes intestinal barrier function by improving mucus and tight junctions in a mouse colitis model. Dig. Liver Dis..

[56] Guan R., Ma N., Liu G., Wu Q., Su S., Wang J., Geng Y. (2023). Ethanol extract of propolis regulates type 2 diabetes in mice via metabolism and gut microbiota. J. Ethnopharmacol..

[57] Ottman N., Geerlings S.Y., Aalvink S., de Vos W.M., Belzer C. (2017). Action and function of Akkermansia muciniphila in microbiome ecology, health and disease. Best Pract. Res. Clin. Gastroenterol..

[58] Wang X., Yan S., Zhao W., Wu L., Tian W., Xue X. (2023). Comprehensive study of volatile compounds of rare Leucosceptrum canum Smith honey: Aroma profiling and characteristic compound screening via GC–MS and GC–MS/MS. Food Res. Int..

[59] Tamanai-Shacoori Z., Smida I., Bousarghin L., Loreal O., Meuric V., Fong S.B., Bonnaure-Mallet M., Jolivet-Gougeon A. (2017). *Roseburia* spp.: A marker of health?. Future Microbiol..

[60] Zhang Z., Zhu T., Li Y., Yu B., Tao H., Zhao H., Cui B. (2025). Butyrate Regulates Intestinal DNA Virome and Lipopolysaccharide Levels to Prevent High-Fat Diet-Related Liver Damage in Rats. J. Agric. Food Chem..

[61] Singh V., Lee G., Son H., Koh H., Kim E.S., Unno T., Shin J.H. (2022). Butyrate producers, “The Sentinel of Gut”: Their intestinal significance with and beyond butyrate, and prospective use as microbial therapeutics. Front. Microbiol..

[62] Shin N.R., Lee J.C., Lee H.Y., Kim M.S., Whon T.W., Lee M.S., Bae J.W. (2014). An increase in the Akkermansia spp. population induced by metformin treatment improves glucose homeostasis in diet-induced obese mice. Gut.

[63] Tengeler A.C., Gart E., Wiesmann M., Arnoldussen I.A.C., van Duyvenvoorde W., Hoogstad M., Dederen P.J., Verweij V., Geenen B., Kozicz T. (2020). Propionic acid and not caproic acid, attenuates nonalcoholic steatohepatitis and improves (cerebro) vascular functions in obese Ldlr^-/-^.Leiden mice. FASEB J..

[64] Scott K.P., Martin J.C., Campbell G., Mayer C.D., Flint H.J. (2006). Whole-genome transcription profiling reveals genes up-regulated by growth on fucose in the human gut bacterium “*Roseburia inulinivorans*”. J. Bacteriol..

[65] Van den Abbeele P., Belzer C., Goossens M., Kleerebezem M., De Vos W.M., Thas O., De Weirdt R., Kerckhof F.M., Van de Wiele T. (2013). Butyrate-producing Clostridium cluster XIVa species specifically colonize mucins in an in vitro gut model. ISME J..

[66] Tolhurst G., Heffron H., Lam Y.S., Parker H.E., Habib A.M., Diakogiannaki E., Cameron J., Grosse J., Reimann F., Gribble F.M. (2012). Short-chain fatty acids stimulate glucagon-like peptide-1 secretion via the G-protein-coupled receptor FFAR2. Diabetes.

[67] An Y., Li Y., Wang X., Chen Z., Xu H., Wu L., Li S., Wang C., Luan W., Wang X. (2018). Cordycepin reduces weight through regulating gut microbiota in high-fat diet-induced obese rats. Lipids Health Dis..

[68] Petriz B.A., Castro A.P., Almeida J.A., Gomes C.P., Fernandes G.R., Kruger R.H., Pereira R.W., Franco O.L. (2014). Exercise induction of gut microbiota modifications in obese, non-obese and hypertensive rats. BMC Genom..

[69] Nair A.B., Jacob S. (2016). A simple practice guide for dose conversion between animals and human. J. Basic Clin. Pharm..

