# Supplementary material for: Propolis Modulates the Gut Microbiota–Gut Hormone–Liver AMPK Axis to Ameliorate High-Fat Diet-Induced Metabolic Disorders in Rats
Source: Nutrients. 2025 Sep 30;17(19):3114. doi: 10.3390/nu17193114 (PMC12525829; doi:10.3390/nu17193114)
Supplement: Supplementary file 1 [file nutrients-17-03114-s001.zip › nutrients-3885171-supplementary.pdf]

Table S1. Primer Sequences

Gene name	Forward Primer	Reserve Primer
Ampk	CTCAGGAAGGCTGTATGCGG	ACGGTTGAGATACTCCGGGAT
Fasn	GCCTAACACCTCTGTGCAGT	GTGAGATGTGCTGCTGAGGT
Sqle	TGCTACAGATGATTCCTTGCGTCAG	ACCCAACAGGACCAGTCAAACATTC
Srebf1	TTTCCTCTTCAACCGGGAGC	CCAATGCCTTTCAGGTCGCA
Acaca	GGAAGTGGAAGGCACAGTGAAGG	CTGCGGATCTGCTTGAGGACATAG
Abcg8	TGTTAAGCCGCTCCCGATAC	GAAGTCCATGGAGCCACACA
Hmgcr	TGGCAGGACGCAACCTCTAC	AATAGTTACCACTGACCGCCAGAA
Hmgcs1	CGGTTCCCTTGCTTCTGTTCTGG	CCTGGTGTGGCATCTTGTGTGAC
Pparα	CGCTGGGTCCTCTGGTTGTC	TTCAGTCTTGGCTCGCCTCT
Apob	TCTGACTGGTGGACTCTGACTGC	TCTTGGAGAGCGTGGAGACTGAC
Cck	CAGGTCCGCAAAGCTCCCT	CCCGGTCACTTATCCTATGGC
Ghrl	TGGGAAGAGGTCAAAGAG	TGTAAGTCAGCAGGAGAG
Gip	TCCTGAGAACCAACAGGTAGGA	CCGAGCCAACAGCTCTTCTTA
Lep	CCAGGATGACACCAAAACCCT	ACCGACTGCGTGTGTGAAAT
Gcg	CGCCAGATCATTCCCAGCTT	CCCTGTGAATGGCGTTTGTC
Ppy	GTACCCGGGGGACTATGCTA	TAGGCCTGGTCAGTGTGTTG
Tjp1	CCATCTTTGGACCGATTGCTG	TCACAGTGTGGCAAGCGTAG
Cldn1	GGCCCTCGATTGCCCTAAAT	AAACCATGTTTGCCTGCGTC
Pparγ	CAGAAACTGGGAGTAGCCTGG	TCTGATCACCAGCAGAGGTC
Muc2	GGCCAGAACTCTACCGGTTC	ATGGCCTTGGAGCAGGTAAC
Gapdh	AACCCATCACCATCTTCCAG	CACGACATACTCAGCACCAG
